# Multiplex malaria antigen detection by bead-based assay and molecular confirmation by PCR shows no evidence of *Pfhrp2* and *Pfhrp3* deletion in Haiti

**DOI:** 10.1186/s12936-019-3010-9

**Published:** 2019-11-27

**Authors:** Camelia Herman, Curtis S. Huber, Sophie Jones, Laura Steinhardt, Mateusz M. Plucinski, Jean F. Lemoine, Michelle Chang, John W. Barnwell, Venkatachalam Udhayakumar, Eric Rogier

**Affiliations:** 1 Malaria Branch, Division of Parasitic Diseases and Malaria, Centers for Disease Control and Prevention, Atlanta, GA USA; 2 CDC Foundation (CDCF), Atlanta, GA USA; 3 Atlanta Research and Education Foundation (AREF), Atlanta, GA USA; 4 U.S. President’s Malaria Initiative, Centers for Disease Control and Prevention, Atlanta, GA USA; 5 Programme National de Contrôle de la Malaria/MSPP, Port-au-Prince, Haiti

**Keywords:** Haiti, Rapid diagnostic test, HRP2 deletion, *Plasmodium* aldolase, *Plasmodium* lactate dehydrogenase, *Pfhrp2*, *Pfhrp3*

## Abstract

**Background:**

The *Plasmodium falciparum* parasite is the only human malaria that produces the histidine-rich protein 2 and 3 (HRP2/3) antigens. Currently, HRP2/3 are widely used in malaria rapid diagnostic tests (RDTs), but several global reports have recently emerged showing genetic deletion of one or both of these antigens in parasites. Deletion of these antigens could pose a major concern for *P. falciparum* diagnosis in Haiti which currently uses RDTs based solely on the detection of the HRP2/3 antigens.

**Methods:**

From September 2012 through February 2014, dried blood spots (DBS) were collected in Haiti from 9317 febrile patients presenting to 17 health facilities in 5 departments throughout the country as part of a bed net intervention study. All DBS from RDT positive persons and a random sampling of DBS from RDT negative persons were assayed for *P. falciparum* DNA by nested and PET-PCR (n = 2695 total). All PCR positive samples (n = 331) and a subset of PCR negative samples (n = 95) were assayed for three malaria antigens by a multiplex bead assay: pan-*Plasmodium* aldolase (pAldo), pan-*Plasmodium* lactate dehydrogenase (pLDH), and HRP2/3. Any samples positive for *P. falciparum* DNA, but negative for HRP2/3 antigens were tested by nested PCR for *Pfhrp2* and *Pfhrp3* gene deletions.

**Results:**

Of 2695 DBS tested for *Plasmodium* DNA, 345 (12.8%) were originally found to be positive for *P. falciparum* DNA; 331 of these had DBS available for antigen detection. Of these, 266 (80.4%) were positive for pAldo, 221 (66.8%) positive for pLDH, and 324 (97.9%) were positive for HRP2/3 antigens. Seven samples (2.1%) positive for *P. falciparum* DNA were not positive for any of the three antigens by the bead assay, and were investigated for potential *Pfhrp2/3* gene deletion by PCR. These samples either successfully amplified *Pfhrp2/3* genes or were at an estimated parasite density too low for sufficient DNA to perform successful genotyping.

**Conclusions:**

Malaria positive samples in multiple Haitian sites were found to contain the HRP2/3 antigens, and no evidence was found of *Pfhrp2/3* deletions. Malaria RDTs based on the detection of the HRP2/3 antigens remain a reliable *P. falciparum* diagnostic tool as Haiti works towards malaria elimination.

## Background

The island of Hispaniola is the only location in the Caribbean region where malaria remains endemic. *Anopheles albimanus* is the primary vector [1], and the primary causative agent is *Plasmodium falciparum* [2, 3] though there is some evidence for the presence of *Plasmodium vivax* [4]. The national policy of the Haitian Ministry of Health is to screen individuals who show symptoms consistent with malaria using one of the World Health Organization (WHO) recommended rapid diagnostic tests (RDTs) [3, 5]. An international consortium (www.malariazero.com) lead by the Haitian Ministry of Health has established a goal to interrupt local transmission of malaria [6–8], and HRP2/3-based RDTs will be a major tool in this endeavor.

Worldwide, malaria RDTs have been in use for nearly 20 years, and they were designed for portability, ease of use and reliability in low resource settings. Demand for RDTs has grown substantially since their initial deployment with an estimated 314 million tests used globally in 2015 [9]. Malaria RDTs can detect *Plasmodium* specific antigens in a small volume (5 to 10 μL) of blood using lateral flow immunochromatography. These tests can be a more feasible alternative to microscopy in many field settings due to their simplicity of use and able to provide a diagnostic result within 20 min [10].

Three proteins with exceptionally-high histidine content are produced by *P. falciparum*: histidine-rich protein 1 (HRP1), HRP2, and HRP3, with HRP2 and HRP3 at the highest expression levels [11, 12]. Due to high rates of production during blood stage infection, the most common target for malaria RDTs is the *P. falciparum*-specific HRP2 antigen, but pan-*Plasmodium* LDH is also a commonly-used target [13–15]. RDTs detecting HRP2 can also react with HRP3 as many antigenic epitopes are shared between the two antigens [16–18]. The HRP2 antigen can remain in a person’s blood for up to 3 months following successful treatment, making it a less reliable true diagnostic for active infection since recent infections would also be detected [14, 19, 20]. Pan-*Plasmodium* LDH and aldolase are proteins that are expressed by all human malaria species, and these proteins are glycolytic enzymes present at relatively high concentrations, but they are cleared from the blood of infected patients within 2 to 7 days of effective drug treatment [20]. Overall, HRP2/3-based RDTs still remain the most popular for field antigen detection since current antibodies available for HRP2/3 have been shown to be more sensitive than anti-aldolase or anti-LDH antibodies [21–23].

The *Pfhrp2* gene is located on the sub-telomeric region of chromosome 8 and contains two exons separated by an intron [24]. Studies interested in the genetic diversity of the HRP2 antigen show extensive variation within isolates of the same country and between isolates of different countries [25, 26]. The *Pfhrp3* gene is approximately 977 base pairs (bp) and is located subtelomerically on chromosome 13 with significant sequence similarity and antigenic cross-reactivity to the same epitopes on the HRP2 antigen [27–29]. Deletion of the *Pfhrp2* and *Pfhrp3* genes can cause false negative RDT test results [30], and recent WHO guidelines state “*a PfHRP2 deletion should be strongly suspected if a patient gives negative result on an HRP2 test line of at least two quality assured malaria RDTs and positive on the pan*- *or PfLDH test line when a combination test is used and when the sample is confirmed microscopically to be positive for P. falciparum by two qualified microscopists*” [31]. In 2010, Peru was the first country to confirm identification of circulating *P. falciparum* parasites with *Pfhrp2/3* deletions, with reports from other countries soon following [26, 32–36]. Malaria parasites are unable to delete the LDH or aldolase antigens as these proteins are essential enzymes for metabolism and survival.

Recently, a bead-based immunoassay platform has been developed for multi-antigen capture and detection with the ability to simultaneously assay for pan-*Plasmodium* and *P. falciparum*-specific antigens [23], and can be used in directly comparing a person’s blood antigen levels with the results obtained from that person’s RDT [9]. Presented here is data from a large-scale screening for symptomatic *P. falciparum* infections in Haiti using PCR assays, and describe the antigen expression profiles for those samples found to be DNA positive for *P. falciparum* malaria, with special consideration given to samples showing a potential profile of deletion for *Pfhrp2/3* genes.

## Methods

### Human subjects

This study was carried out on samples collected for a facility-based, case–control study of bed net efficacy. Participants consented for long-term sample storage (up to 20 years) and additional tests for malaria. As described previously, febrile patients (measured temperature of 37.5 °C or history of fever in past 48 h) were recruited from 17 health facilities in five of the ten Haitian departments [37]. Patient enrollment began in September 2012 and was completed in February 2014. Finger prick samples of blood were taken for malaria RDT (CareStart HRP2; AccessBio, Somerset, NJ) and dried blood spots (DBS) were collected on Whatman 903 cards (GE Healthcare), dried overnight, and stored in a baggie with desiccant until further processing. RDT results were read by study staff and patients with positive malaria tests were treated according to national guidelines. Written informed consent was obtained from patients enrolling in the study. Patients younger than 18 years required consent from a parent or guardian; written assent was also obtained from patients aged 7–17 years. The study protocol was approved by the National Bioethics Committee of Haiti and the Institutional Review Board at the US Centers for Disease Control and Prevention (CDC) in Atlanta, GA, USA.

### Sample preparation and DNA extraction

Upon arrival at CDC, DBS were carefully examined for potential contamination by mold growth and sufficient blood volume (enough for two 6 mm punches) before elution of blood and DNA extraction. For antigen detection, samples were eluted in a blocking buffer (Buffer B: 0.5% Polyvinyl alcohol (Sigma) 0.5% polyvinylpyrrolidine (Sigma), 0.1% casein (ThermoFisher), 0.5% BSA (Sigma), 0.3% Tween-20, 0.05% sodium azide, and 0.01% *E. coli* extract to prevent non-specific binding) to a final concentration of 1:20×. Genomic DNA for molecular analysis was extracted from two 6 mm punches of the filter paper blood spots using the Qiagen DNA extraction kit following the manufacturer’s instructions for blood dried on filter paper (QIAGEN, Valencia, CA). The DNA was eluted in 150 μL of elution buffer, aliquoted, and stored at − 20 °C until further use.

### Antigen multiplex serology

The presence and quantification of antigens was performed with similar methodology as described previously using the bead-based Luminex^®^ platform (Luminex Corp., Austin, TX) [23]. Three unique bead regions (Bio-Plex COOH bead, BioRad, Hercules, CA; 171506XXX) were individually coated by the EDC/Sulfo-NHS intermediate reaction with separate antibodies specific for each antigen to be captured: *Plasmodium* aldolase (12.5 μg/12.5 × 10^6^ beads, rabbit IgG anti-aldolase, Abcam, Cambridge, UK; ab207494), *Plasmodium* LDH (12.5 μg/12.5 × 10^6^ beads, mouse IgG anti-LDH, BBI Solutions, Cardiff, UK; BM355-Z8F7), and *P. falciparum* PfHRP2 (20 μg/12.5 × 10^6^ beads, mouse IgG anti-HRP2, Abcam; ab9206). For the assay, a mix of the three coupled bead regions was made in 5 mL Buffer A (PBS, 0.5% BSA, 0.05% Tween20, 0.02% NaN_3_) so that 1500 of each bead region would be added per well in the assay plate. Samples were incubated with 50 μL of the bead mix in filter bottom plates (Millipore; MABVN1250) for 90 min under gentle shaking and subsequently washed three times with 100 μL wash buffer (PBS, 0.05% Tween20). Beads were incubated for 45 min with a 50 μL mix of detection antibodies: anti-pAldo (1:1000×, rabbit anti-aldolase, Abcam; ab207494), anti-pLDH [1:500× of 2:1:1 mixture (BBI Solutions BM355-P4A2: BioRad Pv-pLDH HCA156: BioRad Pf-pLDH HCA158)], and anti-HRP2 (1:500×, mouse IgG anti-HRP2, Abcam, ab9203). All detection antibodies were previously biotinylated by Thermo Scientific EZ-Link Micro Sulfo-NHS-Biotinylation Kit (ThermoFisher Scientific, Waltham, MA) according to the manufacturer’s protocol. Plates were washed three times, and wells subsequently incubated with 50 μL streptavidin–phycoerythrin (1:200×, Invitrogen, Carlsbad, CA) for 30 min. Plates were washed three times, and after a final 30 min wash step with reagent diluent, beads were washed once and re-suspended in 100 μL PBS and read on a Bio-Plex 200 instrument (BioRad, Hercules, CA**)** by generating the median fluorescence intensity (MFI) signal for 50 beads in each unique region, and then the mean fluorescence intensity of the MFIs among duplicates. The final measure, denoted as MFI-bg, was reported by subtracting MFI values from beads on each plate only exposed to sample diluent during the sample incubation step. As antibodies used in this assay against HRP2 would also recognize the same epitopes present on HRP3, any positive signal for the bead assay using these antibodies is denoted as detection of HRP2/3 since the true signal would not be able to be differentiated between these two antigens from a sample.

To determine an assay signal (MFI-bg signal) which would indicate sample positivity to malaria antigens, a panel of 86 U.S. resident blood samples were run by both the multiplex antigen assay (at 1:20 dilution) in order to obtain the mean and standard deviation for a “malaria non-exposed” population. For each antigen, the mean + 3 s.d. MFI-bg value for this non-exposed population was used at the positivity threshold. To extrapolate an assay signal to an antigen concentration, standard curves of known recombinant antigens were run in order to generate equations to derive a concentration from a signal intensity of the bead assay [23]. Recombinant pLDH and HRP2 antigens were provided by Microcoat Biotechnologie GmbH (Bernried, Germany), and lyophilized preparations were rehydrated according to the manufacturer’s instructions. The *Plasmodium vivax*-specific isoform of aldolase was produced at the CDC as described previously [23].

All *P. falciparum* PCR-positive samples were tested by the multiplex antigen assay (n = 331), and an additional 95 PCR-negative samples were also randomly chosen for antigen testing. In the initial study, two samples were found to be *P. vivax* PCR positive [37] and were also assayed for malaria antigens.

### PCR assays to screen for *Plasmodium* DNA

As described in the initial publication [37], of 9317 patients enrolled in the study, PCR screening was performed on samples from all RDT-positive persons providing a valid DBS (n = 331) as well as a randomly-chosen subset of DBS from RDT-negative persons (n = 2363) for a total of 2695 samples undergoing PCR screening. Following DNA extraction, PET-PCR was performed as described previously [38]. Briefly, PET-PCR reaction mix consisted of: 10 μL of 2× ABI Taq Man Buffer (Thermo Fisher Scientific), 0.5 μL of 10 μM genus forward and reverse primers (reverse primer bound to FAM dye), 0.5 μL of 10 μM *P. falciparum* forward primer, 0.25 μL of 10 μM *P. falciparum* reverse (bound to HEX dye) primer, 6.25 μL of nuclease-free water (Ambion, Thermo Fisher Scientific) for a total reaction mixture to 18 μL/sample. To each reaction tube, 2 μL of extracted DNA was added. Real-time PCR was performed on a Stratagene Mx3005p machine (Agilent, Santa Clara, CA) with thermal cycling conditions of 95 °C for 10 min followed by 45 cycles of 95 °C for 40 s and 60 °C for 10 s. Cycle threshold (Ct) values above 40 were considered to be malaria DNA negative and Ct values below 40 considered to be parasitemia positive [38]. Samples with any Ct value had results confirmed by nested PCR (nPCR) for 18S DNA as described previously [39] with the nPCR result considered the final determination for positivity to *Plasmodium* DNA.

### PCR assays for *Pfhrp2* and *Pfhrp3* genotyping

Samples were further investigated for potential deletion of the *Pfhrp2* and *Pfhrp3* genes if they were positive to *P. falciparum* DNA by PCR assays, but not found to be positive for HRP2/3 antigen by the bead assay. Nested PCR was performed on these samples under conditions described previously [40] with *Pfmsp1* and *Pfmsp2* genes to validate DNA quality. Reaction buffer and polymerase enzyme came from Sigma-Aldrich High-Fidelity PCR system kit. Primer sets for all nPCR reactions are shown in Additional file 1, thermocycling conditions in Additional file 2, and nPCR reaction mixtures in Additional file 3. nPCR products were stained by Gel Red Nucleic Acid gel stain (Biotum, Fremont, CA) and visualized by gel electrophoresis in a 2% agarose gel. DNA size standards were separated alongside PCR products to allow sizing of species and control bands.

### Statistical analysis

Parametric (logistic) and non-parametric (LOESS) dose–response regression curves were made in R software version 3.3.0 using the stats package (R Foundation for Statistical Computing, Vienna, Austria) [9].

## Results

**Fig. 1:**
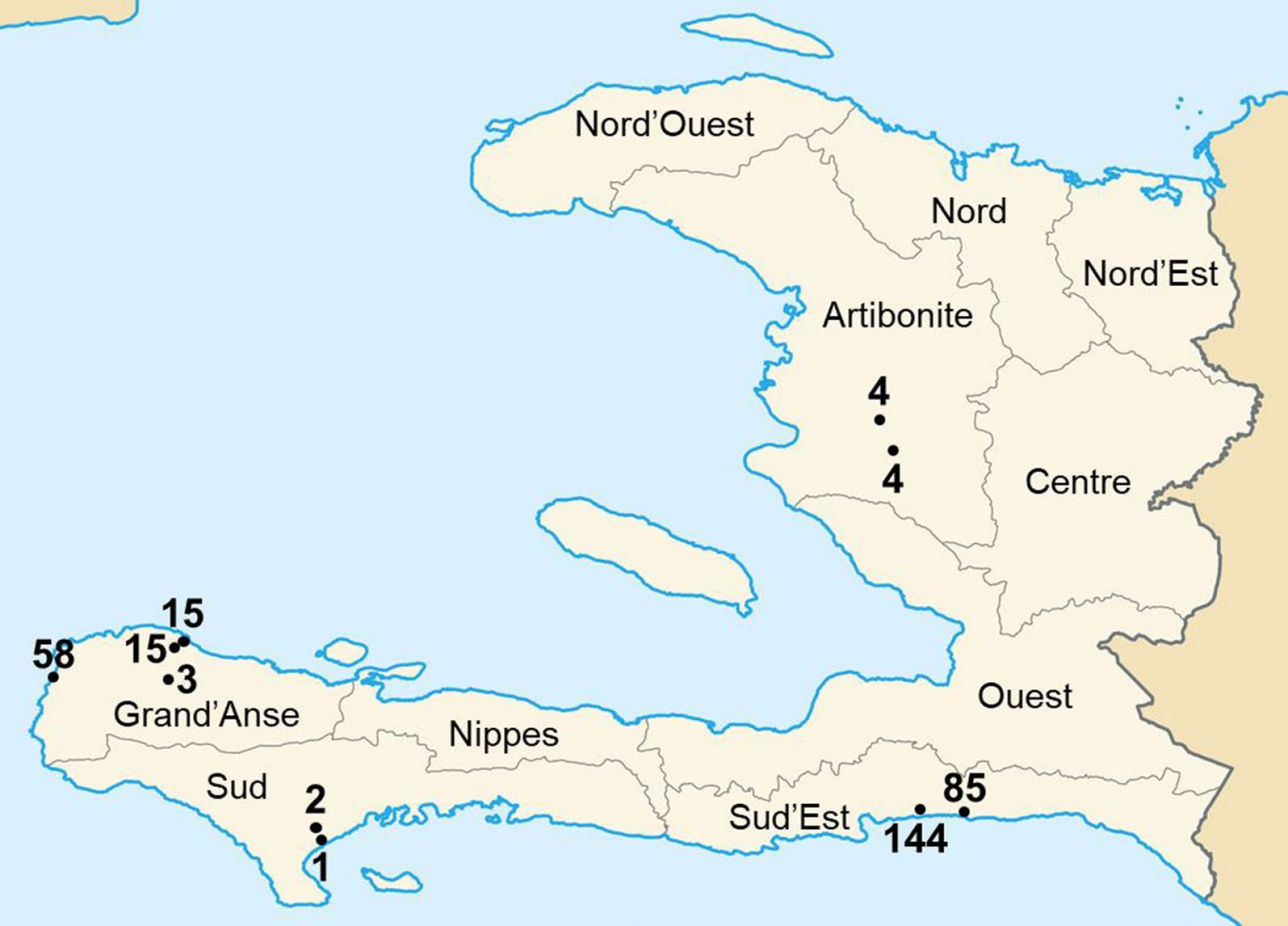
Departments of Haiti and locations of health facilities providing the 331 *P. falciparum* positive dried blood spots. Health facilities shown as dots on the map, and number of PET-PCR positive samples from each facility indicated by the number

**Fig. 2:**
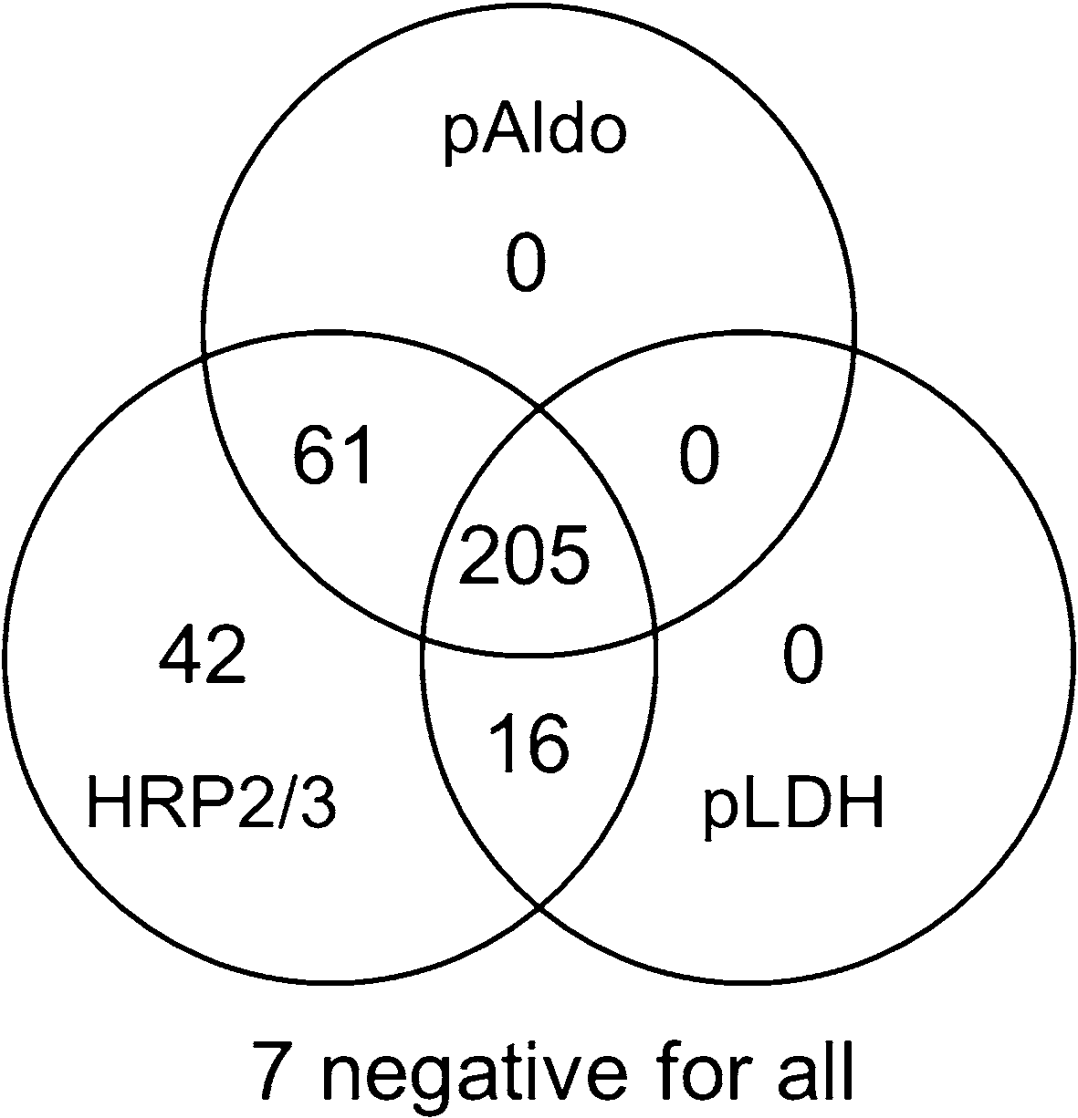
Different profiles of antigen positivity for 331 samples found to be PCR positive for *P. falciparum* DNA. Numbers indicate concordance of antigen positivity for all samples

Complete sample workflow for the study is shown in Additional file 4. As previously reported, of the 2695 samples screened for malaria DNA, 345 were PCR positive [37]. Of these, 331 had DBS available for multiplex antigen detection of pAldo, pLDH and HRP2/3. Figure 1 shows the location of the ten health facilities from the four departments where these 331 samples originated, and number of samples per health facility. Of 331 PCR positive samples with available DBS, 266 (80.4%) were positive for pAldo antigen, 221 (66.8%) positive for pLDH, and 324 (97.9%) were positive for HRP2/3, with samples showing different multi-antigen profiles (Fig. 2). No PCR-positive samples were positive for the pan-*Plasmodium* antigens pAldo or pLDH without also being positive for the HRP2/3 antigen. Two samples found to be PCR positive for *P. vivax* by the initial study were assayed for multiplex antigen detection with one sample showing positivity to the pLDH and HRP2/3 antigens and one sample positivity to only the HRP2/3 antigen. Of the 95 PCR-negative samples chosen, 3 (3.2%) showed positivity to any antigens: two samples positive for only HRP2/3, and one positive to both pAldo and HRP2/3.

**Fig. 3:**
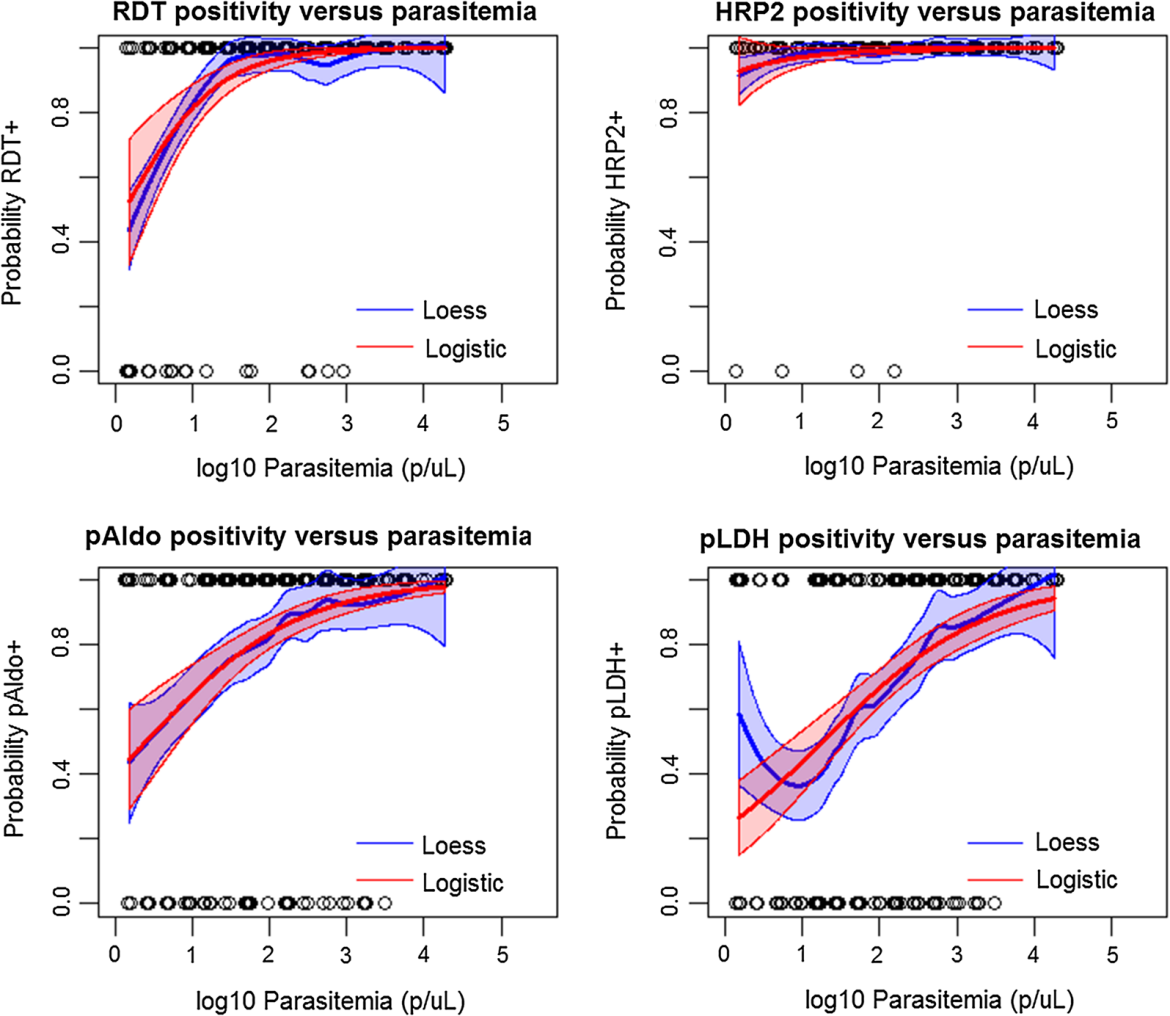
RDT and antigen positivity as a function of PCR-determined parasite density. Non-parametric (LOESS, blue lines) and parametric (logistic, red lines) regression of probability of single antigen positivity as a modeled by estimated parasite density. Outputs at selected probabilities displayed in Table 1

**Fig. 4:**
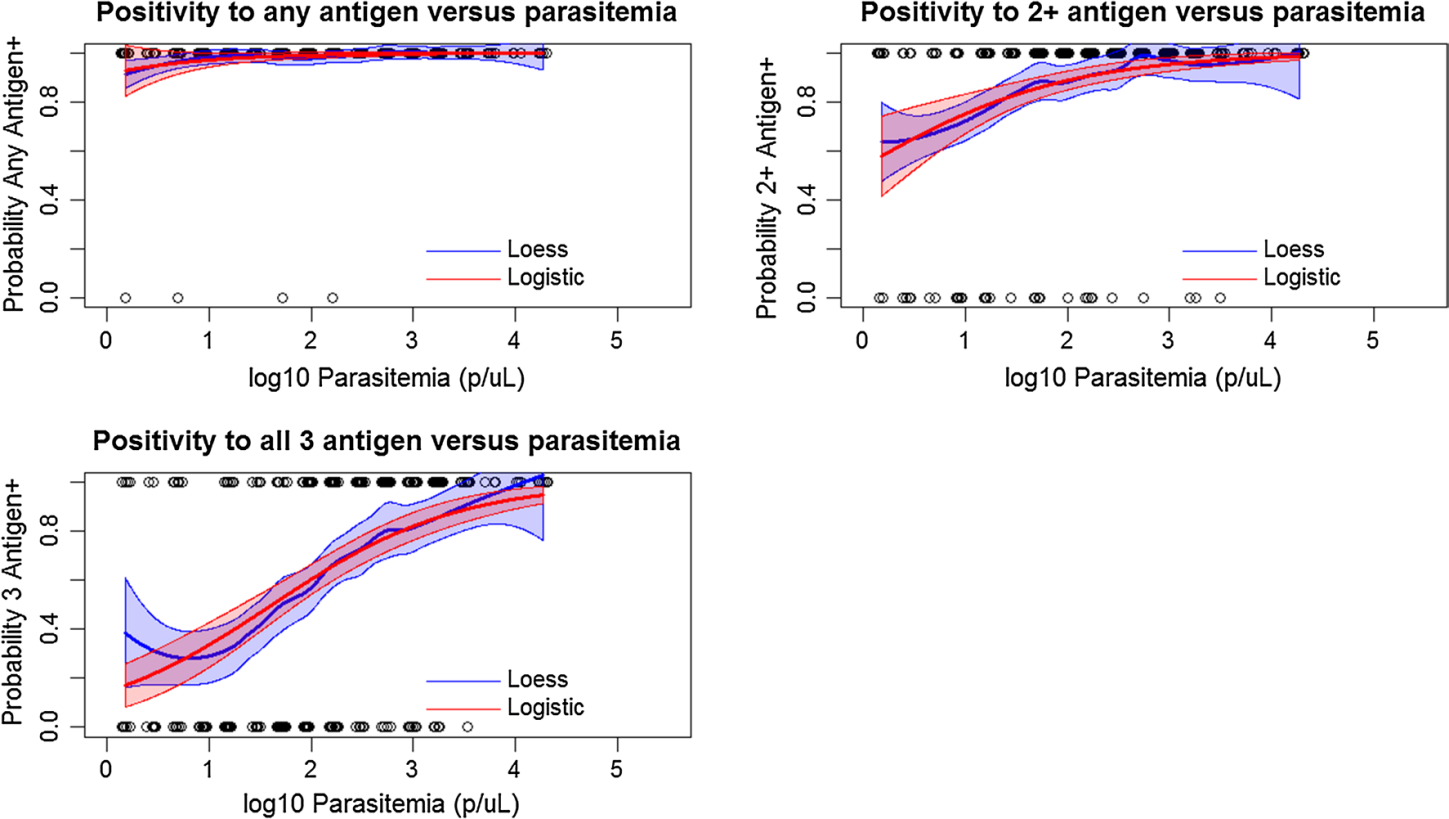
Positivity of combinations of antigen positivity as a function of parasite density. Non-parametric (LOESS, blue lines) and parametric (logistic, red lines) regression of probability of antigen positivity as a modeled by estimated parasite density. Outputs at selected probabilities displayed in Table 1

Figure 3 illustrates non-parametric (LOESS regression) and parametric (logistic regression) for RDT or antigen positivity as a function of estimated parasite density by PET-PCR. As the estimated parasite density for a person’s blood sample decreases, both parametric and non-parametric curves predict lower probabilities of being antigen positive—for both RDT detection and the bead assay. At a parasite density of 77.5 p/μL blood, the RDTs used in this survey were found to have a 95% probability of being positive (Table 1). At the 95% criteria of antigen positivity, the same estimates were 2056 p/μL for pAldo, and 3.2 p/μL for HRP2/3 antigens. The 95% probability criteria was unable to be estimated for pLDH by the logistic curve. Figure 4 shows the breadth of antigen positivity (i.e., whether a sample is positive, for 1, 2, or all 3 antigens) by the bead assay as a function of PET-PCR estimated parasite density. At the 95% probability criteria, positivity to any one antigen was found to be at an estimated 8.3 p/μL, two antigens at 285.3 p/μL, and all three antigens at 8909 p/μL. Table 1 displays the logistic regression estimates for antigen positivity as a function of estimated parasite density by PCR.

**Table 1:** Sensitivity of different malaria tests as a factor of parasite density or antigen concentration

Test	Probability of test positivity			
	50%	75%	90%	95%
Parasite density thresholds (Pf p/μL)				
HRP2 RDT+	–	6.1 (2.1–11)	27.7 (12–53)	77.5 (31–177)
Bead-based HRP2+	–	–	–	3.2 (1.5–130)
Bead-based pAldo+	2.5 (1.5–6.7)	30.8 (12–60)	377.1 (147–1073)	2056 (520–8527)
Bead-based pLDH+	18.3 (6.6–37)	266.1 (135–576)	3901 (1190–14,589)	–
Parasite density thresholds (Pf p/μL)				
Positivity to any one antigen	1.7 (1.3–2.2)	3.0 (2.2–4.4)	5.5 (3.3–8.5)	8.3 (4.1–13)
Positivity to any two antigens	6.7 (4.5–9.8)	27.3 (18–42)	110.2 (60–190)	285.3 (130–540)
Positivity to all three antigens	57.1 (37–87)	375.0 (224–648)	2464 (1119–5210)	8909 (3109–NA)
HRP2 concentration thresholds (HRP2 pg/mL)				
HRP2 RDT+	561.5 (305–899)	1609 (925–2472)	4548 (2438–7806)	9346 (4185–17,432)

*HRP2* histidine-rich protein 2, *RDT* rapid diagnostic test, *pAldo* pan-*Plasmodium* aldolase, *pLDH* pan-*Plasmodium* lactate dehydrogenase

**Fig. 5:**
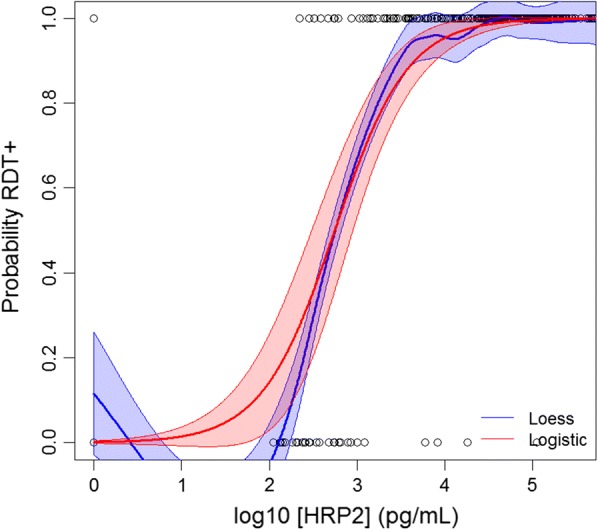
Sensitivity of HRP2-based RDT as a function of HRP2 concentration. Non-parametric (LOESS, blue line) and parametric (logistic, red line) regression of probability of RDT positivity as a modeled by HRP2 antigen concentration in person’s blood sample. Outputs at selected probabilities displayed in Table 1

HRP2-based RDT results reflect the amount of the HRP2/3 antigen in a person’s blood at the time of sampling, regression modeling can also predict at what blood concentration of HRP2/3 RDTs used in a survey were reliably giving a positive test result [9]. Of 428 samples (PCR positives and negatives) tested by the bead assay for multiplex antigen detection, 424 had RDT results and 294 (69.3%) came from persons with a positive RDT result. Of the 294 recorded RDT positives, 293 (99.7%) of the DBS samples from these persons were positive for the HRP2/3 antigen assay. Figure 5 shows regression modeling for the HRP2-based RDT positivity as a function of HRP2/3 concentration, and estimates at different probability levels. At 95% probability of a positive RDT result, the HRP2/3 concentration in the person’s blood sample was modeled to be 9346 pg/mL. This is lower than the 95% probability estimate of 41,000 pg/mL reported from a previous study in Haiti [9].

Of all 331 PCR-positive DBS samples, 7 (2.1%) were not positive for any of the three antigens by the bead assay. Since PCR-positive samples would be predicted to be positive for at least HRP2/3 [41], if not also pAldo and pLDH [23], these seven samples were further investigated for potential deletions of the *Pfhrp2* and *Pfhrp3* genes. Nested PCRs were performed for all of these samples with targets for both of the *Pfhrp2* and *Pfhrp3* exon-1–2 spanning segments as well as exon 2. The additional single-copy genes of *Pfmsp1* and *Pfmsp2* were amplified to confirm integrity of the sample DNA [24, 40]. One of the seven samples had shown amplification of *Pfhrp2* and *Pfhrp3* genes, but gave negative results for pAldo, pLDH and HRP2/3 antigens in the bead assay, despite three repeats. The remaining 6 samples were estimated to be very low parasite density infections, with one of approximately 51 p/μL and the remaining under 5 p/μL. Likely due to the low concentration of DNA in these samples, *Pfmsp1* and *Pfmsp2* were unable to be amplified, and thus, the *Pfhrp2/3* genotyping results could not be reported with confidence.

## Discussion

Hispaniola is the only remaining island in the Caribbean with endemic malaria, and more than 95% of all cases in Hispaniola come from Haiti [7]. Prior to 2010, confirmed malaria diagnosis in Haiti was primarily performed by microscopy, but after the 2010 earthquake, RDTs started to be deployed throughout the country and set the groundwork for a permanent adoption of RDTs as the primary method of malaria diagnosis [3]. Post-earthquake, the Haitian government in collaboration with the CDC approved RDTs targeting only the HRP2/3 antigen since *P. falciparum* is the predominant malaria species in the country. This is the first study that examines evidence of *Pfhrp2/3* gene deletion in Haiti; the evidence was generated using both molecular and antigen detection tools for appropriate characterization.

No evidence was found for *P. falciparum* parasites with deletions of both the *Pfhrp2* and *Pfhrp3* genes in Haiti. Of all samples *P. falciparum* infection identified by PET-PCR, 49.8% had a concentration of HRP2/3 of 50,000 pg/mL or higher showing the high concentration of the HRP2/3 antigens in these symptomatic infections. Additionally, no PET-PCR *P. falciparum*-positive samples were found to be positive for the pan-*Plasmodium* antigens (pLDH and pAldo), but negative for HRP2/3 antigens. A similar methodology was employed for a recent study in Angola by showing the ability of the multiplex antigen assay for use as a screening tool to discern if samples are lacking the HRP2/3 antigens, but have pan-*Plasmodium* antigens present [23]. Practically, this scenario is explained by the presence of infection with only non-falciparum malaria parasites, or infection with a strain of *P. falciparum* not expressing the HRP2 and HRP3 antigens. Since the HRP2 and HRP3 antigens are abundantly expressed by wild-type *P. falciparum* [14, 19], PCR-identified infections would be expected to have HRP2/3 antigen present. In this current study, of all the PET-PCR positive samples, 7/331 (2.1%) of the samples showed absence of all the three antigens, but were largely confirmed to be very low-density infections with molecular data unable to be reported since other single-copy genes could not be amplified [40]. Additionally, 6 of these 7 persons tested RDT negative during the field survey. This finding shows the difficulty of accurate reporting and genotyping of low-density infections since so little antigen and DNA would be present, likely below the limit of detection for the laboratory assays being used. As has been suggested previously, reporting of potential *Pfhrp2* and *Pfhrp3* deletions is likely most appropriate from symptomatic and higher parasite density infections [24].

Unlike many countries in the South American region, the country of Haiti does not appear to harbor *Pfhrp2*- or *Pfhrp3*-deleted parasites. Clinical cases of *Pfhrp2* gene deletion were first reported in Peru’s Amazonian region [36], followed by Suriname [27], Brazil [42], Bolivia [42], and Colombia [28, 43]. Low prevalence of HRP2-negative parasites has also been reported in numerous other global locations across different continents [23, 32, 33]. With the majority of RDT tests worldwide using the HRP2/3 protein as a determinant factor in malaria diagnosis, this potential for deletions of the HRP2/3 antigen causes much concern in public health officials and healthcare settings worldwide where *P. falciparum* is endemic. As deletion of HRP2/3 proteins will lead to false negative test results, such individuals may not be treated and this will lead to increased morbidity and mortality rates, while also enabling deleted parasites to continue to seed transmission to the local area [44]. Though this report did not find evidence of deletions in Haiti, continual monitoring would be beneficial as Haiti moves towards malaria elimination and continues to rely on RDTs for clinical diagnosis. Attractive options for gathering data on potential deletions may come from community or health facility surveys which are already performing microscopy, PCR-based tests, or other non-HRP2/3 antigen confirmation for detection of *P. falciparum* positive persons. Known *P. falciparum* positive blood samples could then be screened for the presence of HRP2/3 antigens by sensitive laboratory assays.

This study has several important limitations. As the only sample type was blood dried on filter paper, the potential exists for antigen or DNA degradation over time. Additionally, the locations used in this study do not represent the *P. falciparum* genotypes of the entire country since no facilities were included from six of the ten Haitian departments. Future surveillance should aim to gather samples from more sites in the country.

## Conclusion

Deletions of the *P. falciparum* genes for the HRP2 and HRP3 antigens can compromise the performance of HRP2/3-based RDTs and adversely affect malaria healthcare delivery in a nation like Haiti which relies strongly on RDTs for detection of *P. falciparum* infection. The data presented here shows no evidence of deletion in both the *Pfhrp2* and *Pfhrp3* genes in Haiti, giving strong support for the continued use of HRP2/3-based RDTs for detection of *P. falciparum* infection in Haiti.

## Supplementary information

**Additional file 1.** Nested PCR Primers for Genes *Pfhrp2*, *Pfhrp3*, *Pfmsp1* and *Pfmsp2*.

**Additional file 2.** Nested PCR Thermocycling Conditions for Genes *Pfhrp2*, *Pfhrp3*, *Pfmsp1* and *Pfmsp2*.

**Additional file 3.** Nested PCR Reaction Mixtures for Genes *Pfhrp2*, *Pfhrp3*, *Pfmsp1* and *Pfmsp2*.

**Additional file 4.** Flowchart for sample workflow.

## Data Availability

All the data generated by this study are included in the manuscript. Raw data is available upon reasonable request.

## Abbreviations

DBS: dried blood spot
DNA: deoxyribonucleic acid
HRP2: histidine rich protein 2
HRP3: histidine rich protein 3
pAldo: pan-*Plasmodium* aldolase
PET-PCR: photo-induced electron transfer polymerase chain reaction
pLDH: pan-*Plasmodium* lactate dehydrogenase
RDT: rapid diagnostic test
