# Supplementary material for: Multiplex malaria antigen detection by bead-based assay and molecular confirmation by PCR shows no evidence of Pfhrp2 and Pfhrp3 deletion in Haiti
Source: Malar J. 2019 Nov 27;18:380. doi: 10.1186/s12936-019-3010-9 (PMC6882344; doi:10.1186/s12936-019-3010-9)
Supplement: Supplementary file 1 — Additional file 1. Nested PCR Primers for Genes Pfhrp2, Pfhrp3, Pfmsp1 and Pfmsp2. [file 12936_2019_3010_MOESM1_ESM.docx]

**Additional file 1. Nested PCR Primers for Genes *Pfhrp2*, *Pfhrp3*, *Pfmsp1* and *Pfmsp2***

| **Target** | **nPCR**  **Reaction** | **Forward Primer**  **(5’-3’)** | **Reverse Primer**  **(5’-3’)** |
| --- | --- | --- | --- |
| ***Pfhrp2* (exon 1-2)** | Primary | GGT-TTC-CTT-CTC-AAA-AAA-TAA-AG | TCT-ACA-TGT-GCT-TGA-GTT-TCG |
| ***Pfhrp3* (exon 1-2)** | Primary | GGT-TTC-CTT-CTC-AAA-AAA-TAA-AA | CCT-GCA-TGT-GCT-TGA-CTT-TA |
| ***Pfhrp2* (exon 2)** | Primary | TTC-CGC-ATT-TAA-TAA-TAA-CTT-GTG | GGC-AAT-GTG-TGG-CGG-CTT |
| ***Pfhrp3* (exon 2)** | Primary | CTC-CGA-ATT-TAA-CAA-TAA-CTT-GTT | AGT-GAT-GCG-TAG-TGG-CAT-TA |
| ***Pfmsp1*** | Primary | CTA-GAA-GCT-TTA-GAA-GAT-GCA-GTA-TTG | ATT-CTA-ATT-CAA-GTG-GAT-CAG |
| ***Pfmsp2*** | Primary | GAA-GGT-AAT-TAA-AAC-ATT-GTC | GAT-GTT-GCT-GCT-CCA-CAG |
| ***Pfhrp2* (exon 1-2)** | Secondary | GTA-TTA-TCC-GCT-GCC-GTT-TTT-GCG | CTA-CAC-AAG-TTA-TTA-TTA-AAT-GCG-GAA |
| ***Pfhrp3* ( exon 1-2)** | Secondary | ATA-TTA-TC-GCT-GCC-GTT-TTT-GCT | CTA-AAC-AAG-TTA-TTG-TTA-AAT-TCG-GAG |
| ***Pfhrp2* (exon 2)** | Secondary | CGA-AAC-TCA-AGC-ACA-TGT-AGA | CTT-CGT-GGT-GTG-CGG-CTG |
| ***Pfhrp3* (exon 2)** | Secondary | GAA-AGT-CAA-GCA-CAT-GCA-GG | GCA-TTA-TGG-TGT-GCT-CCA-TC |
| ***Pfmsp1*** | Secondary | CTA-GAA-GCT-TTA-GAA-GAT-GCA-GTA-TTG | CAT-ATC-CAT-CAA-TTA-AAT-ATT-TGA-AAC-C |
| ***Pfmsp2*** | Secondary | GAG-TAT-AAG-GAG-AAG-TAT-G | CTA-GAA-CCA-TGA-ATA-TGT-CC |
