# Supplementary material for: Multiplex malaria antigen detection by bead-based assay and molecular confirmation by PCR shows no evidence of Pfhrp2 and Pfhrp3 deletion in Haiti
Source: Malar J. 2019 Nov 27;18:380. doi: 10.1186/s12936-019-3010-9 (PMC6882344; doi:10.1186/s12936-019-3010-9)
Supplement: Supplementary file 2 — Additional file 2. Nested PCR Thermocycling Conditions for Genes Pfhrp2, Pfhrp3, Pfmsp1 and Pfmsp2. [file 12936_2019_3010_MOESM2_ESM.docx]

**Additional file 2. Nested PCR Thermocycling Conditions for Genes *Pfhrp2*, *Pfhrp3*, *Pfmsp1* and *Pfmsp2*.**

| **Target** | **nPCR Reaction** | **Initial denature - denature/annealing/elongating - final extension** | **Number of cycles** |
| --- | --- | --- | --- |
| ***Pfhrp2* (exon 1-2)** | Primary | 95°C;5 min - 95°C;30s/55°C;30s/68°C;30s - 68°C;5min | x30 |
| ***Pfhrp3* ( exon 1-2)** | Primary | 95°C;5 min - 95°C;30s/53°C;30s/68°C;30s - 68°C;5min | x30 |
| ***Pfhrp2* (exon 2)** | Primary | 95°C;5 min - 95°C;30s/55°C;30s/68°C;1min - 68°C;5min | x30 |
| ***Pfhrp3* (exon 2)** | Primary | 95°C;5 min - 95°C;30s/55°C;30s/68°C;1min - 68°C;5min | x30 |
| ***Pfmsp1*** | Primary | 95°C;5 min - 95°C;30s/51°C;30s/68°C;1min - 68°C;5min | x30 |
| ***Pfmsp2*** | Primary | 95°C;5 min - 95°C;30s/50°C;30s/68°C;1min - 68°C;5min | x30 |
| ***Pfhrp2* (exon 1-2)** | Secondary | 95°C;5 min - 95°C;30s/62°C;30s/68°C;30s - 68°C/5min | x30 |
| ***Pfhrp3* ( exon 1-2)** | Secondary | 95°C;5 min - 95°C;30s/62°C;30s/68°C;30s - 68°C/5min | x30 |
| ***Pfhrp2* (exon 2)** | Secondary | 95°C;5 min - 95°C;30s/57°C;30s/68°C;1min - 68°C/5min | x20 |
| ***Pfhrp3* (exon 2)** | Secondary | 95°C;5 min - 95°C;30s/57°C;30s/68°C;1min - 68°C/5min | x20 |
| ***Pfmsp1*** | Secondary | 95°C;5 min - 95°C;30s/52°C;30s/68°C;1min - 68°C/5min | x30 |
| ***Pfmsp2*** | Secondary | 95°C;5 min - 95°C;30s/48°C;30s/68°C;1min - 68°C/5min | x30 |
