# Supplementary material for: Multiplex malaria antigen detection by bead-based assay and molecular confirmation by PCR shows no evidence of Pfhrp2 and Pfhrp3 deletion in Haiti
Source: Malar J. 2019 Nov 27;18:380. doi: 10.1186/s12936-019-3010-9 (PMC6882344; doi:10.1186/s12936-019-3010-9)
Supplement: Supplementary file 3 — Additional file 3. Nested PCR Reaction Mixtures for Genes Pfhrp2, Pfhrp3, Pfmsp1 and Pfmsp2. [file 12936_2019_3010_MOESM3_ESM.docx]

**Additional file 3. Nested PCR Reaction Mixtures for Genes *Pfhrp2*, *Pfhrp3*, *Pfmsp1* and *Pfmsp2*.**

| **Reaction Mix for nested PCR** | ***Pfhrp2* (exon 1-2) primary rxn** | ***Pfhrp2* (exon 1-2) secondary rxn** | ***Pfhrp3* (exon 1-2) primary rxn** | ***Pfhrp3* (exon 1-2) secondary rxn** |
| --- | --- | --- | --- | --- |
| **10x HF* buffer** | 2 µl | 2 µl | 2 µl | 2 µl |
| **dNTPs** | 2 µl | 2 µl | 2 µl | 2 µl |
| **forward primer (15µM)** | 1 µl | 1 µl | 1 µl | 1 µl |
| **reverse primer (15µM)** | 1 µl | 1 µl | 1 µl | 1 µl |
| **HF* enzyme** | .2 µl | .2 µl | .2 µl | .2 µl |
| **Nuclease- free water** | 11.8 µl | 11.8 µl | 11.8 µl | 11.8 µl |
| **DNA** | 2 µl | 2 µl^a^ | 2 µl | 2 µl^a^ |
| **Total rxn** | **20 µl** | **20 µl** | **20 µl** | **20 µl** |

| **Reaction Mix for nested PCR** | ***Pfhrp2* (exon 2) primary rxn** | ***Pfhrp2* (exon 2) secondary rxn** | ***Pfhrp3* (exon 2) primary rxn** | ***Pfhrp3* (exon 2) secondary rxn** |
| --- | --- | --- | --- | --- |
| **10x HF* buffer** | 2 µl | 2 µl | 2 µl | 2 µl |
| **dNTPs** | 2 µl | 2 µl | 2 µl | 2 µl |
| **forward primer (15µM)** | 1 µl | 1 µl | 1 µl | 1 µl |
| **reverse primer (15µM)** | 1 µl | 1 µl | 1 µl | 1 µl |
| **HF* enzyme** | .2 µl | .2 µl | .2 µl | .2 µl |
| **Nuclease- free water** | 11.8 µl | 11.8 µl | 11.8 µl | 11.8 µl |
| **DNA** | 2 µl | 2 µl^b^ | 2 µl | 2 µl^b^ |
| **Total rxn** | **20 µl** | **20 µl** | **20 µl** | **20 µl** |

| **Reaction Mix for nested PCR** | ***Pfmsp1* primary rxn** | ***Pfmsp1* secondary rxn** | ***Pfmsp2* primary rxn** | ***Pfmsp2* secondary rxn** |
| --- | --- | --- | --- | --- |
| **10x HF* buffer** | 2 µl | 2 µl | 2 µl | 2 µl |
| **dNTPs** | 2 µl | 2 µl | 2 µl | 2 µl |
| **forward primer (15µM)** | 1 µl | 1 µl | 1 µl | 1 µl |
| **reverse primer (15µM)** | 1 µl | 1 µl | 1 µl | 1 µl |
| **HF* enzyme** | .2 µl | .2 µl | .2 µl | .2 µl |
| **Nuclease- free water** | 11.8 µl | 12.8 µl | 11.8 µl | 12.8 µl |
| **DNA** | 2 µl | 1 µl | 2 µl | 1 µl |
| **Total rxn** | **20 µl** | **20 µl** | **20 µl** | **20 µl** |

* HF: high-fidelity reagents from Sigma Aldrich kit; ^a^2µL of a 1:10 dilution of the primary product; ^b^2µL of a 1:200 dilution of the primary product.
