# Supplementary figures and images for: Multiplex malaria antigen detection by bead-based assay and molecular confirmation by PCR shows no evidence of Pfhrp2 and Pfhrp3 deletion in Haiti
Source: Malar J. 2019 Nov 27;18:380. doi: 10.1186/s12936-019-3010-9 (PMC6882344; doi:10.1186/s12936-019-3010-9)

**Additional file 4. Flowchart for sample workflow.**

**
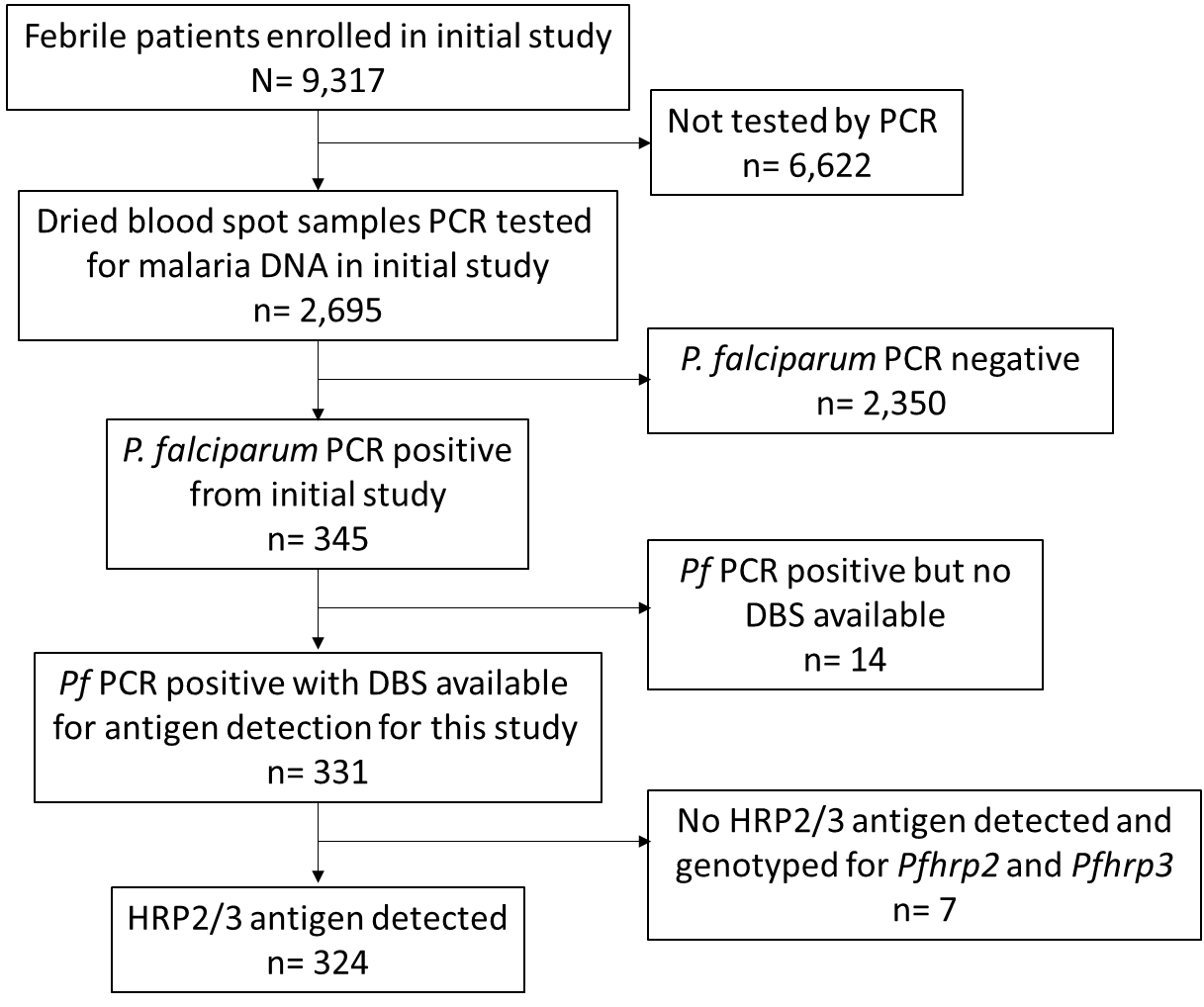
**

Supplement: Supplementary file 4 — Additional file 4. Flowchart for sample workflow. [file 12936_2019_3010_MOESM4_ESM.docx]
